# Botulism Associated with Home-Fermented Tofu in Two Chinese Immigrants — New York City, March–April 2012

**Published:** 2013-07-05

**Authors:** Edward Chai, Eugene Choi, Cristina Guitierrez, Melvin Hochman, Suja Johnkutty, Wael Kamel, Todd Mekles, Reza Zarnegar, Joel Ackelsberg, Sharon Balter, Ellen H. Lee, Lan Li, Angel Ramos, Teresa Rodriguez, Don Weiss, Janette Yung, Benyang Zhao, Stephen W. Davis, Christina Egan, George E. Hannett, Agam Rao, Amita Toprani, Nandini Sreenivasan

**Affiliations:** New York Hospital, Queens; New York City Dept of Health and Mental Hygiene; New York State Dept of Health; Div of Foodborne, Waterborne, and Environmental Diseases, National Center for Emerging and Zoonotic Infectious Diseases; EIS officers, CDC

In March 2012, the New York City Department of Health and Mental Hygiene (DOHMH) received two reports of recent immigrants from China admitted to the same hospital 23 days apart for suspected foodborne botulism. Patient 1 had a laboratory-confirmed case of foodborne botulism, and patient 2 had a probable case; patient 1’s case was definitively associated with home-fermented tofu, and patient 2’s case might have been associated with home-fermented tofu. Both patients had purchased fresh tofu from the same Chinese grocery in Queens, a New York City borough, in January 2012, and each had prepared home-fermented tofu using similar recipes. Similar fermentation practices at the two homes might have facilitated toxin production. Testing confirmed botulinum toxin type B in home-fermented tofu consumed by patient 1. Bulk tofu at the grocery in Queens was found to be sold in unrefrigerated, uncovered, water-filled bins. Traceback revealed that the grocery’s fresh bulk tofu supplier at the time of the patients’ purchases had gone out of business. DOHMH advised the grocery’s manager of the need to properly store bulk tofu. Public health responders and clinicians should be aware of the association between botulism and fermented tofu.

## Patient 1

On March 3, 2012, a Chinese man aged 39 years arrived at the hospital with a 4-day history of vomiting followed by dysphagia, diplopia, dysarthria, dyspnea, and difficulty walking. Neurologic examination revealed bilateral cranial nerve deficits: dilated pupils minimally reactive to light, ptosis, oculomotor palsy, and facial paralysis. Motor strength was normal, but deep tendon reflexes were hypoactive. He was admitted to the intensive-care unit and intubated because of concern for impending respiratory failure. An edrophonium chloride test was interpreted as positive for myasthenia gravis, and intravenous immune globulin treatment was initiated. Electromyography studies eventually were determined to be suspicious for, but not diagnostic of, botulism. On March 9, unilateral upper extremity weakness was noted, and results of a test for antibodies to acetylcholine receptors (positive in myasthenia gravis) were negative. Serum and stool specimens were obtained for testing, and botulinum antitoxin was administered. On March 27, botulinum toxin type B was identified by mouse bioassay in stool specimens. Patient 1 improved and was discharged to a rehabilitation facility on March 26.

## Patient 2

On March 28, 2012, a Chinese woman aged 36 years from the same Queens neighborhood as patient 1 was admitted to the same hospital after 2 days of vomiting and diarrhea followed by dysarthria, dysphagia, and dizziness. On examination, she had bilateral cranial nerve palsies: ptosis, dilated pupils minimally reactive to light, and oculomotor palsy. Mild, right upper extremity weakness and loss of upper extremity deep tendon reflexes were noted. She was intubated because of concern regarding impending respiratory failure. The same clinicians who had cared for patient 1, and who by this time had laboratory confirmation of botulism in patient 1, admitted patient 2; they immediately suspected botulism because of the similar clinical presentation. On March 29, serum and stool specimens were obtained, and botulinum antitoxin was administered. Electromyography studies performed March 30 were consistent with, but not diagnostic of, botulism. No botulinum toxin was detected in serum or stool specimens. The patient improved and was discharged home on April 18.

## Public Health Investigation

On February 25 and 26, approximately a week before symptom onset, patient 1 and his wife ate home-fermented tofu prepared by patient 1’s wife. Patient 1’s wife consumed the same amount of tofu as patient 1, but was asymptomatic. Patient 2 consumed home-fermented tofu on at least 3 of the 7 days preceding symptom onset. No other persons were known to have eaten patient 2’s tofu.

Patient 1’s wife and patient 2 had emigrated from the same locality in Jiangxi Province, China, to the United States within the previous 2 years. Both resided in Queens, but they did not know each other. They reported purchasing fresh bulk tofu in January 2012 at the same Chinese grocery in Queens. Patient 1’s wife cubed the tofu and placed it in a plastic container in layers separated by heavy paper. She covered the container with a nonairtight lid and allowed the contents to ferment at room temperature for 1 week. She next added chili pepper and salt, transferred the tofu to a glass jar, and stored it in the refrigerator for 3 weeks before consumption. Patient 2 placed blocks of tofu in a colander covered with plastic wrap and kept it at room temperature for 7–10 days. She then added salt, dried chili pepper, and orange peel, and stored the fermented tofu in glass jars in the refrigerator. The fermented tofu was not heated before consumption in either case.

On March 29, samples of fermented tofu were collected from both patients, and fresh bulk tofu was obtained from the grocery for laboratory testing. No samples of unfermented tofu purchased by the patients in January were available for testing. The laboratory detected botulinum toxin type B by mouse bioassay on April 2 in leftover fermented tofu from the same batch consumed by patient 1. Toxin was not detected in tofu from patient 2, in any additional foods from either household, or in fresh tofu obtained by DOHMH from the grocery in March 2012.

To help detect additional cases, DOHMH notified health-care practitioners and issued press releases in English and Chinese; no new cases were identified. A site visit to the grocery revealed that bulk tofu was sold in unrefrigerated, uncovered, water-filled bins. DOHMH informed the manager that bulk tofu must be maintained at a temperature <41°F (<5°C) in covered or sneeze-guard–protected containers in a well-supervised area with a means of preventing bare-hand contact. Food traceback revealed that the grocery’s fresh bulk tofu supplier at the time of the patients’ purchases had since permanently closed, and the business owner no longer resided in the United States.

### Editorial Note

This suspected outbreak included one confirmed case linked to consumption of home-fermented tofu (patient 1) and one probable case in a person who also ate home-fermented tofu (patient 2). The recognition of these cases prompted concern that other cases might follow, and a rapid and vigorous public health response was conducted. This investigation was challenging because both clinical presentations were atypical, and because fermented tofu, an uncommon vehicle for botulism in the United States, was not immediately recognized as the potential source of illness.

Botulism typically causes bilateral cranial nerve palsies, followed by bilateral descending flaccid paralysis over the course of hours or days, with eventual loss of deep tendon reflexes. Patients are usually afebrile, and sensation and cognition are unaffected; in foodborne botulism, neurologic symptoms might be preceded by nausea and vomiting (1,2). Atypical presentations, such as those of both patients in this cluster, can make recognition of botulism difficult. Both patients eventually were determined to have bilateral cranial nerve deficits, but this was not initially clear. Both patients also had loss of deep tendon reflexes and respiratory compromise but minimal or no muscle weakness. In patient 2’s case, the weakness and loss of reflexes were unilateral. Patient 1 also had a positive edrophonium chloride test, a finding indicative of myasthenia gravis and only rarely reported positive in botulism (3). The clinicians caring for patient 2 ruled out other diseases that have similar signs and symptoms, and electromyography results were consistent with, but not diagnostic of, botulism.

Foodborne botulism occurs when *Clostridium botulinum* spores, which are ubiquitous in the environment, germinate and produce toxin. Spore germination and toxin formation require warm, anaerobic environments with low-acid, low-salt, and low-sugar content (4). A patient’s history of exposure to foods commonly associated with botulism can help with recognition of botulism. In the United States, home-canned foods and traditional fermented Alaska Native foods are major sources of botulism (5). Fermented tofu has only once been reported as associated with botulism in the United States (6). In China, however, home-fermented tofu and other fermented bean products cause the majority of foodborne botulism cases (7). The occurrence of two suspected cases in such close temporal and geographic proximity increased suspicion of a common vehicle, although patient 1’s tofu was the only confirmed source of botulinum toxin; no other foods tested from either household were determined to be a toxin source.

What is already known on this topic?

Foodborne botulism is caused by eating foods contaminated with botulinum toxin produced by the bacterium *Clostridium botulinum*. Botulism is characterized by acute onset of bilateral cranial nerve palsies followed by descending symmetric flaccid paralysis that can progress to respiratory failure or death. In the United States, foodborne botulism typically is associated with home-canned foods and traditional fermented Alaska Native foods.

What is added by this report?

This report highlights the potential for consumption of home-fermented tofu, a food commonly prepared in Chinese communities, to be a risk factor for botulism in the United States. It also documents the atypical clinical presentation of one confirmed and one probable case of botulism from home-fermented tofu prepared from fresh tofu purchased at the same grocery.

What are the implications for public health practice?

Public health professionals should be aware of the association between fermented tofu and botulism, and that botulism can present atypically. Early recognition of botulism can lead to timely diagnosis and appropriate treatment of suspected cases.

Contamination of bulk tofu with *C. botulinum* spores might have occurred at the tofu manufacturing facility or at the grocery. Both patients had purchased tofu during the same month from the same grocery and fermented it using similar recipes. Subsequently, the fermentation processes, which involved prolonged storage at room temperature in a low-acid and low- salt environment, might have created conditions conducive to spore germination and toxin formation. Neither patient heated the tofu before eating it; therefore, toxin would not have been inactivated by heat. Neither patient reported using an airtight container for fermentation, but anaerobic pockets might have existed within the tofu. Previous investigations reveal that botulinum toxin can be distributed unevenly in food (8), which might explain why patient 1’s wife did not contract botulism. Uneven distribution of toxin also might explain the negative test results for patient 2’s leftover tofu.

Public health responders and clinicians should be aware that fermented foods, including tofu, can be vehicles for foodborne botulism. They should consider botulism as the potential cause of cranial nerve palsies and ask about consumption of foods known to cause botulism. Education of populations known to include fermented tofu in their diets might help prevent foodborne botulism associated with consumption of home-fermented tofu.
